# Comparative analysis of the nutritional quality of plant-based and traditional meat products across European markets

**DOI:** 10.3389/fnut.2025.1603600

**Published:** 2025-07-16

**Authors:** Cristina Filip, Béla Kovács, Tibor Casian, Amalia Miklos, Paula Nistor Pop, Tudor-Ionuț Istrate, Victoria Hodoroga, Amelia Tero-Vescan

**Affiliations:** ^1^Department F1, Biochemistry and Environmental Chemistry, George Emil Palade University of Medicine, Pharmacy, Science and Technology of Târgu Mureș, Târgu Mureș, Romania; ^2^Department of Pharmaceutical Technology and Biopharmacy, “Iuliu Hațieganu” University of Medicine and Pharmacy, Cluj-Napoca, Romania; ^3^Department ED1, Economic Studies, Faculty of Economy and Law Departments, George Emil Palade University of Medicine, Pharmacy, Science and Technology of Târgu Mureș, Târgu Mureș, Romania; ^4^Faculty of Medicine, George Emil Palade University of Medicine, Pharmacy, Science and Technology of Târgu Mureș, Târgu Mureș, Romania; ^5^Department ME1 Chemistry and Medical Biochemistry, George Emil Palade University of Medicine, Pharmacy, Science and Technology of Târgu Mureș, Târgu Mureș, Romania

**Keywords:** plant-based meat, nutritional quality, dietary transition, market differences, public health

## Abstract

**Introduction:**

The increasing global demand for plant-based meat (PBM) alternatives highlights the need for comprehensive assessments of their nutritional quality in comparison to traditional meat products.

**Methods:**

This study evaluates the nutritional profiles of PBM and meat products from major supermarket chains in Romania, Germany, and Ireland. The analysis focused on key nutritional parameters, including energy value, macronutrient composition, and fiber content.

**Results:**

PBM products exhibited a lower energy density, reduced saturated fat content, and significantly higher fiber levels than their meat counterparts. However, protein content remained lower in PBM products, while salt levels varied by category. Notably, products from Romania displayed inferior nutritional profiles compared to those from Germany and Ireland, with higher energy, fat, and salt content but lower fiber levels.

**Conclusion:**

These findings underscore the need for policy-driven improvements in PBM formulations and standardized nutritional guidelines across markets. The study contributes to the growing body of research on sustainable dietary transitions and their implications for public health.

## 1 Introduction

The market for plant-based meat (PBM) products is projected to reach €34 billion by 2027 according to the Polaris Market Research (1). The current market trend focuses on the continuous development of new products, with 4,965 PBM items launched globally in the past 5 years. In Germany alone, 391 new products were introduced, positioning the country as a leader in this sector in terms of both sales value and growth rate. However, according to the Smart Protein Report, Romania has also experienced an increase in PBM sales (2).

PBM analogues are predominantly vegetarian or vegan products designed to closely resemble meat in sensory properties. Unlike traditional vegetarian or vegan alternatives, these products are formulated not only for consumers following a plant-based diet but also for flexitarians and omnivores seeking to reduce their consumption of animal products (3, 4). By replicating the texture, color, and taste of conventional meat, PBMs aim to appeal to individuals accustomed to the sensory experience of animal-based foods (5). Consequently, these products offer a viable alternative for omnivores and flexitarians who wish to integrate more plant-based options into their diets while maintaining familiar sensory attributes (4, 6).

Globally, the transition toward a diet that incorporates more plant-based meat (PBM) products and reduces meat consumption is increasingly encouraged. These dietary recommendations stem from several pressing global concerns, including population growth, the expansion of large-scale livestock farming—linked to public health risks and rising antibiotic resistance—environmental degradation, and animal welfare issues. Additionally, the COVID-19 pandemic has influenced consumer perceptions of meat consumption due to the heightened awareness of zoonotic disease risks (7–9). Plant-based diets are widely recognized for their potential to reduce mortality risk factors, particularly ischemic heart disease (10), while also contributing to improved blood pressure regulation (11, 12) and diabetes management (13, 14). PBMs have gained popularity as a more sustainable alternative to conventional meat, offering lower levels of saturated fats and no cholesterol, which can support cardiovascular health (15). Moreover, PBM products are often rich in fiber, vitamins, and minerals, contributing to overall well-being. However, concerns exist regarding their level of processing, as many PBM products contain additives, high sodium levels, and artificial ingredients, which may counteract some of their health benefits (16). In contrast, traditional meat—particularly red and processed varieties—is associated with an increased risk of cardiovascular diseases (17, 18), certain cancers (19), and other health complications (20) due to its saturated fat content and the formation of carcinogenic compounds during cooking (19). The growing interest in sustainable protein alternatives also aligns with rising concerns about certain health risks, including the increasing incidence of colorectal cancer, for which meat consumption has been identified as a contributing factor. According to the International Agency for Research on Cancer (IARC), all types of muscle meat—including beef, veal, pork, and lamb—are classified as “probably carcinogenic” (Group 2A). Additionally, processed meats, which undergo treatments such as curing, smoking, or the addition of preservatives to enhance flavor and shelf life, are classified as “carcinogenic to humans” (Group 1) (21, 22). While PBMs offer a promising alternative, consumers are advised to prioritize minimally processed options to maximize their nutritional and health benefits.

Beyond the need for sustainable alternative protein sources, another critical aspect of novel foods is their nutritional value, which can vary depending on the specific food legislation of each country. Regulations governing the nutritional content of food products differ significantly worldwide, reflecting diverse national priorities related to public health and consumer protection (23). For instance, some countries such as France, Belgium, Germany have implemented stringent food labeling requirements, mandating detailed disclosures on calories, fat, carbohydrates, and other key nutrients. Other countries like Denmark prioritize the restriction or prohibition of certain ingredients, such as trans fats, sodium nitrite, or monosodium glutamate (24). Additionally, food safety and health standards vary considerably, with some nations enforcing strict limits on food additives and contaminants, while others, such as Romania, maintain more flexible regulations regarding additives and labeling practices. Despite these regulatory differences, there is a growing global movement toward greater food transparency and consumer health protection. As scientific research advances and public awareness increases, food legislation continues to evolve to address emerging health concerns and industry developments (25, 26). In the European Union (EU), the most widely used food labeling system is Nutri-Score. This system assigns a score and color rating (ranging from A to E and green to red) to indicate the nutritional quality of a food product. Several European countries, including Germany, have already adopted Nutri-Score. In Romania, Nutri-Score is not yet mandatory; however, ongoing discussions within the EU aim to harmonize food labeling systems at a European level. Future policy developments at both the European and national levels may influence the adoption of Nutri-Score or a similar system in Romania, potentially leading to increased regulatory pressure for its implementation (27). The consumption of alternative meat products in Romania remains lower compared to other EU countries (28). This discrepancy is largely influenced by the high cost of these products, their limited availability in supermarkets, and a general lack of consumer awareness. To promote the adoption of sustainable protein sources, it is essential to enhance public awareness regarding their benefits and the reasons for increasing their consumption (29, 30).

In the past decade, a rising number of scientific articles has focused on comparing animal-based and plant-based foods regarding their nutritional compositions. In a recent study published by Petersen and Hirsch meat and meat alternatives were compared for their nutritional value in five major European countries, including France, Germany, UK, Italy, and Spain (31). Also, current research trends are also focusing on the assessment of the market changes for meat products and plant-based meat alternatives as published by Lindberg et al. (32). In a global comparison of meat and plant-based alternatives, European countries like Lithuania, Germany, Hungary are enlisted amongst the highest consumers of traditional meat, whereas Vietnam leads in plant-based meat substitutes, followed closely by the UK and Hong Kong (33, 34). The plant-based meat sector is expanding, with Germany taking the lead in Europe. India and China demonstrate greater acceptance of plant-based and clean meat options compared to the USA (35). Compared to Western European countries such as Germany, the Netherlands, and the UK—where plant-based alternatives are widely accepted and experiencing rapid growth—Eastern Europe trails in both market penetration and consumer acceptance. Nevertheless, the region holds strong growth potential as awareness rises, and infrastructure continues to develop.

Although several studies have been published in recent years assessing the nutritional profile of PBMs (3, 8, 36–38), further research is needed to compare equivalent products across different countries and determine whether significant nutritional differences exist (39, 40). Moreover, to better understand the dynamics at play, further research is needed into market development, consumer behavior, regulatory policies, and public health planning, to evaluate the similarities and differences across countries with varying geographic and socio-economic contexts.

The aim of this study was to statistically analyse the nutritional value of plant-based meat (PBM) products compared to traditional meat products available in major supermarkets in Romania, Germany, and Ireland, as the consumption of plant-based meat (PBM) alternatives is highly encouraged, given future concerns regarding the sustainability of animal protein sources. To the authors’ knowledge this is the first study focusing on comparing the nutritional value of meat products and meat alternatives in Romania.

## 2 Materials and methods

### 2.1 Samples and data collection

A cross-sectional study focusing on the nutritional composition of traditional meat products and PBM alternatives designed to imitate meat was conducted to identify similarities and differences between these foods. The products were selected from major supermarket chains in Romania (Kaufland, Lidl, Carrefour, Auchan), Germany (ALDI, NORMA, Netto, REWE, EDEKA, Kaufland, Lidl, PENNY), and Ireland (Tesco, SuperValu, Dunnes Stores, Lidl, ALDI). These retail chains were chosen due to their widespread presence, ensuring a representative sample of products available to the general population. To enhance the reliability of our sample, we prioritized large supermarkets over smaller stores.

For each product, brand name, descriptive name, ingredient list, and nutritional composition data were collected. The extracted nutritional parameters included energy value (kcal/100 g), saturated fat (g/100 g), unsaturated fat (g/100 g), carbohydrates (g/100 g), sugars (g/100 g), protein (g/100 g), fiber (g/100 g), and salt (g/100 g). Additionally, we recorded the presence of specific components such as minerals, vitamins, sodium nitrite, monosodium glutamate, natural color additives, artificial color additives, and other additives.

Product identification and data collection were performed in-store between November 2022 and March 2023, ensuring that all analyzed products were physically available to consumers at the time of the study. Each supermarket location was systematically screened—particularly the refrigerated and frozen food sections dedicated to meat products and meat alternatives.

### 2.2 Selection criteria of food products and categories

The products analyzed in this study were preselected based on their formulation to closely replicate the sensory and structural characteristics of conventional meat products, using plant-based ingredients. Only items explicitly marketed with meat-associated terms (e.g., ‘burger’, ‘sausage’, ‘bacon’, ‘minced meat’, ‘meatballs’) and designed to serve as direct analogues to traditional meat were included. Products not intended to mimic meat—such as conventional vegetarian or vegan items (e.g., falafel, tofu blocks, lentil patties, vegetable fritters) were excluded to ensure consistency and relevance in the comparative nutritional analysis. This selection approach is consistent with that of other studies published in the scientific literature (3, 8), which have focused on comparable PBM categories to assess nutritional quality across different markets.

### 2.3 Statistical analysis

The on-pack information for both meat and plant-based meat products was entered into Microsoft Excel®, including details such as brand name, country of origin, nutritional content per 100 g (covering energy value, macro- and micro-nutrients, and additives). If a product was available from both retailers, it was recorded only once in the database. Products were then categorized by their country of origin and product type. Following each grouping, a random sample of approximately 10% of the products was cross verified against the original packaging to ensure data completeness.

Outlier values detected by Grubb’s test were identified, although retained in the statistical analysis to analyse the real samples existing on different markets. This approach ensures that the statistical analysis captures the complete range of variability found in real samples, offering a more accurate reflection of market conditions.

The normal distribution of datasets was evaluated by the Kolmogorov–Smirnov test to detect data skewness and was considered as a fundamental test for choosing the downstream parametric or non-parametric statistical analysis. Pairwise comparison of normally distributed data was evaluated using unpaired t-test, whereas non-Gaussian data were analyzed using the Mann–Whitney U-test.

Statistical analysis between different groups was evaluated using one-way ANOVA with Tukey’s post-hoc test or Kruskal-Wallis test with Dunn’s post-hoc test for normal and non-normal distributed data, respectively. Statistical analysis was carried out using GraphPad InStat 3.06 software (GraphPad Software Inc., San Diego, USA). Statistical significance was considered at *p* < 0.05. Further analysis of class differences was evaluated using the SIMCA 17 software (Sartorius Stedim Biotech, Göttingen, Germany). OPLS—DA models (Orthogonal partial least squares discriminant analysis) were created to identify similarities and differences between the studied products in terms of nutritional components.

The first set of models aimed to compare animal meat and PBM products (all products and separately on product category), while the second model aimed to identify nutritional patterns of different groups identified by hierarchical clustering analysis. Both X (nutrients data) and Y (dummy variable matrix reflecting class membership) were scaled to unit variance before model fitting. Loading and score plots were generated for interpretation purposes. It is especially valuable for identifying patterns in complex datasets, such as those encountered in food quality studies. The advanced features of the software enable a detailed examination of class differences and help to achieve a thorough understanding of the nutritional components across various products and markets.

## 3 Results

### 3.1 Product selection and overall nutrient profile

Nutritional data were gathered from 330 products across three countries with varying levels of economic development and purchasing power (Table 1). In terms of labeling, the majority of PBM products carried a vegan certification, with a few exceptions classified as vegetarian due to the inclusion of egg-derived ingredients. Other common claims on PBM packaging included “gluten-free” and “source of fiber.” The products were categorized into five groups—sausages, burgers, minced meat, meatballs, and bacon—and analyzed statistically to identify correlations between different variables.

**Table 1:** Categories of products analyzed in the study.

Category	Features for PBM products	PBM products (*n* = 160)	Meat products (*n* = 170)
Burgers	Products designed to replicate meat and labeled with the term “burger”	46	48
Sausages	Products designed to replicate meat and labeled with the term “sausages”	41	50
Meatballs	Balls designed to resemble meat in appearance and texture.	45	25
Minced meat	A product designed to resemble “minced meat” in appearance and texture.	16	24
Bacon	Strips designed to replicate the appearance and texture of “bacon.”	12	23

As expected, most of the tested variables showed a non-normal distribution of the data. Therefore, the pairwise comparison of the variables collected for meat and PBM products was performed using non-parametric evaluation (Mann–Whitney test). Statistically significant (*p* < 0.0001) class differences between meat products and PBM product were observed for all categories, except for the salt content (*p* = 0.625). Meat products were generally characterized by higher energy value (*μ* ≈ 245 kcal/100 g), having approximately 20% more energy compared to PBM products (*μ* ≈ 200 kcal/100 g), a difference that was statistically significant (*p* < 0.0001). This difference in energy values was primarily attributed to the higher saturated and unsaturated fat content of meat products. The mean saturated fat content of meat products was found to be nearly three times higher than that of PBM products (*p* < 0.0001), whereas the unsaturated fat content was 25% higher in meat products compared to PBM products (*p* = 0.0001). Furthermore, protein content was significantly greater in meat products, with an average 30% increase compared to PBM counterparts (*p* < 0.0001). In contrast, PBM products presented notably higher carbohydrate, sugar and fiber content. Carbohydrate content in PBM products was 3.5 times higher (*p* < 0.0001), and the sugar content was more than double that found in meat products (*p* < 0.0001). As anticipated, the fiber content of PBM products was substantially elevated, being 14 times greater than that of meat products (*p* < 0.0001). The descriptive statistical indicators for different variables in the case of meat and PBM products are listed in Table 2.

**Table 2:** Descriptive statistics of meat and PBM products

Nutritional content	Energy value (kcal/100 g)	Saturated fats (g/100 g)	Unsaturated fats (g/100 g)	Carbohydrates (g/100 g)	Sugars (g/100 g)	Fibers (g/100 g)	Proteins (g/100 g)	Salt (g/100 g)
Meat products (*n* = 170)								
D	0.071 *p* = 0.03	0.104 *p* < 0.001	0.083 *p* < 0.01	0.237 *p* < 0.0001	0.217 *p* < 0.0001	0.384 *p* < 0.0001	0.064 *p* = 0.07	0.060 *p* > 0.10
Average	245.3	7.4	11.1	2.9	0.8	0.3	17.1	1.5
Min	113.0	0.8	1.1	0.0	0.0	0.0	0.5	0.1
Q1	209.2	5.3	7.5	0.4	0.3	0.0	14.0	0.9
Median	240.5	7.7	10.6	1.1	0.5	0.0	17.4	1.5
Q3	283.0	8.7	14.0	4.3	1.0	0.4	20.0	2.0
Max	463.0	20.0	29.0	13.0	9.7	14.0	31.2	4.5
IQR	73.8	3.4	6.5	3.9	0.7	0.4	6.0	1.1
PBM products (*n* = 160)								
D	0.043 *p* > 0.10	0.235 *p* < 0.0001	0.054 *p* > 0.10	0.146 *p* < 0.0001	0.161 *p* < 0.0001	0.122 *p* < 0.0001	0.157 *p* < 0.0001	0.104 *p* < 0.001
Average	200.6	2.5	8.7	10.2	1.9	4.3	12.8	1.5
Min	56.0	0.1	0.1	0.0	0.0	0.0	2.0	0.7
Q1	167.8	0.9	5.0	5.0	0.8	2.3	7.6	1.1
Median	201.0	1.3	8.9	7.7	1.6	4.5	12.0	1.4
Q3	234.0	3.5	11.6	14.4	2.8	5.8	16.0	1.8
Max	366.0	14.0	24.9	30.0	7.4	15.0	91.0	4.3
IQR	66.3	2.6	6.6	9.4	2.0	3.5	8.4	0.7
*p*	<0.0001	<0.0001	0.0001	<0.0001	<0.0001	<0.0001	<0.0001	0.652

n—number of products, D—statistical value of distribution and related probability results, Q—quartile, IQR—interquartile range, p—statistical difference for nutritional values between PBM and meat products.

**Figure 1:**
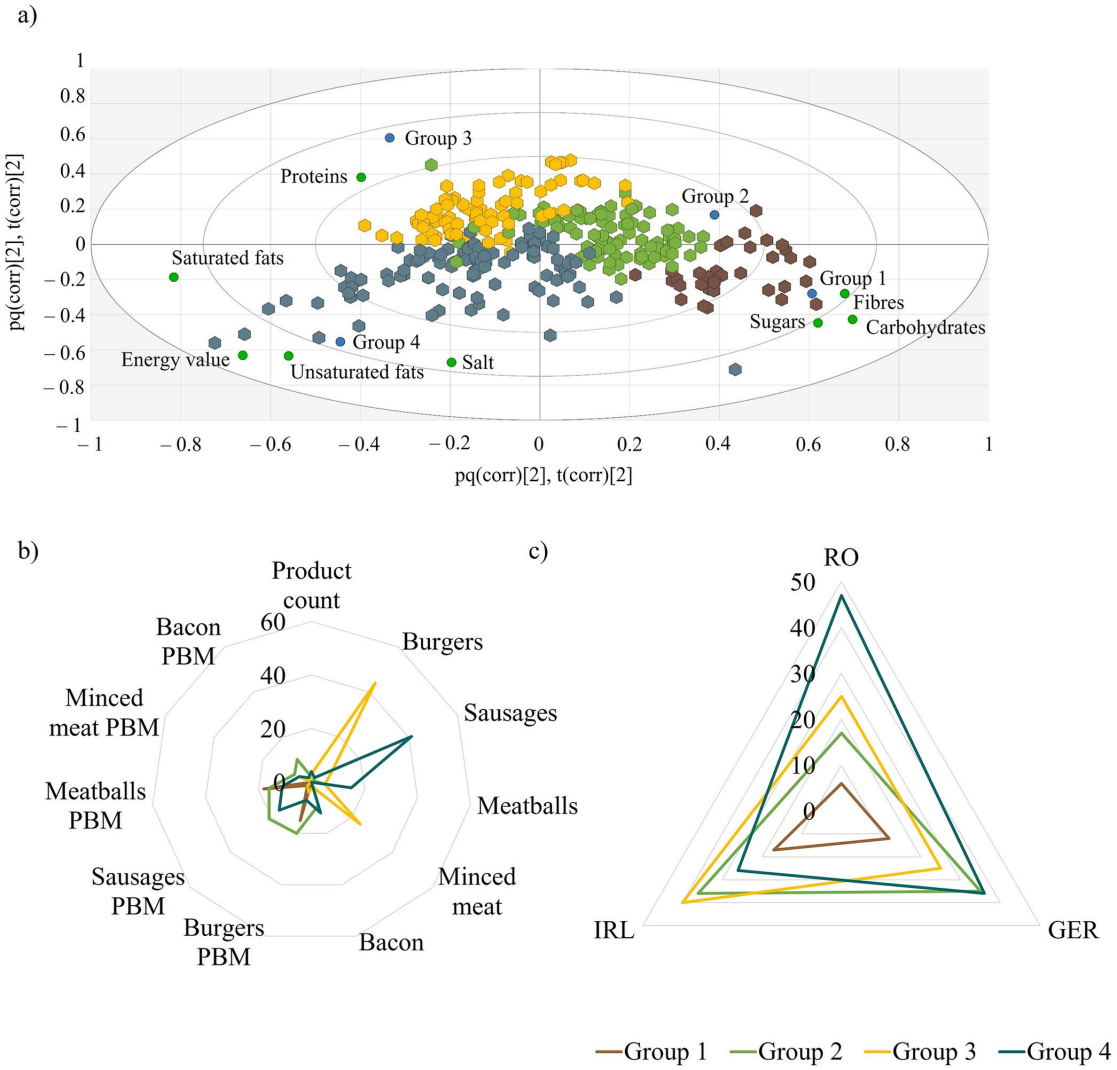
**(a)** Biplot of the four-way clustering of meat and PBM products, green circles indicate nutritional components, brown hexagons—Group 1, green hexagons—Group 2, yellow hexagons—Group 3 and blue hexagons—Group 4 of animal meat and PBM products as resulted from the hierarchical clustering analysis. The “pq” and “t” indicate the co-charting of scores and loadings of the data to display similarities and differences between observations; **(b)** SeDeM plot of product distribution according to data clustering in terms of nutritional components; **(c)** SeDeM plot of product distribution according to data clustering in terms of nutritional component by countries.

To identify similarities and differences between all products regardless of source and processing, a PCA-X based hierarchical clustering analysis was employed. Clustering of data was based on the scores of the PCA model built on nutrient data (Figures 1a–c).

Group 1 (Figure 1a, brown hexagons) was exclusively characterized by PBM type products. Thirty-five products—burgers and meatballs—of this nature were the most abundant in fibers, carbohydrates, and sugars. Almost half of the products listed in this category were from the Irish market (48.6%), followed by Germany (34.3%), and finally Romania (17.1%). The products belonging to Group 3 (Figure 1a, yellow hexagons) were mainly characterized by a conspicuous protein content where 83 meat and only 10 PBM type products were categorized. Regarding the market distribution, 44.4% of the products were identified from Ireland, whereas products found on the Romanian and German markets shared an equal of 27.8% of these.

The disparities between Group 1 and Group 3 in terms of nutritional values and market shares were bridged by products belonging to Group 2 (Figure 1a, green hexagons). This group was rather characterized by the particularities of PBM products. However, some products were also good sources of proteins. Group 2 was characterized by 91 products of which the ratio of PBM vs. animal meat was 4:1. In terms of market distribution, 80% of the products were found in Germany or Ireland in equal distribution, whilst once again the Romanian market seemed substandard with only 20% of the products found on this Eastern European market. Group 4 (Figure 1a, blue hexagons) were mainly characterized by higher fat content and implicitly had greater energy values compared to the other groups. Furthermore, the salt content of representatives from group 2 was elevated compared to the rest of the clusters.

This array of products was the most heterogenous in terms of representatives from both PBM and animal meat categories. Of a total of 121 products, 41 PBM and 70 classical meat products were listed in this cluster. Conversely to the previous groups, products from Romania were the most expressed in this array with 43.1% being from this market. One-third of the products were identified on the German market, whereas only 23.9% in Ireland.

### 3.2 Statistical analysis of nutritional composition of products by category

Supplementary Table 1 gives a detailed statistical analysis of the main nutrients for all the products for each category studied. For meat products, the energy values lie between 209.3 and 295.0 kcal/100 g, higher than values obtained for their PBM counterparts of 181.8–218.8 kcal/100 g product. This difference, as disclosed, is driven by the higher lipid content; however, it is important to mention that even though the saturated and unsaturated fat content of the meat products are higher, discrepancies are more obvious in the case of saturated fats, which, besides energy service, account for health benefits as well. The PBM products are richer in fibers with values ranging from 3.5 to 5.2 g/100 g compared to 0.0–0.8 g/100 g on average found in meat products, which is also reflected in higher carbohydrate and sugar content. Even though the protein content of meat products is superior to their PBM counterparts this is not as conspicuous as in the case of fibers. The protein content of meat products ranges from 14.5 to 18.9 g/100 g product, while for PBM type products from 11.2 to 16.0 g/100 g product. The salt content is comparable between different products with values in average of 1.40 g/100 g (0.2–2.5 g/100 g) for meat products and 1.56 g/100 g (1.3–2.1 g/100 g) for meat alternatives.

#### 3.2.1 Burgers

**Figure 2:**
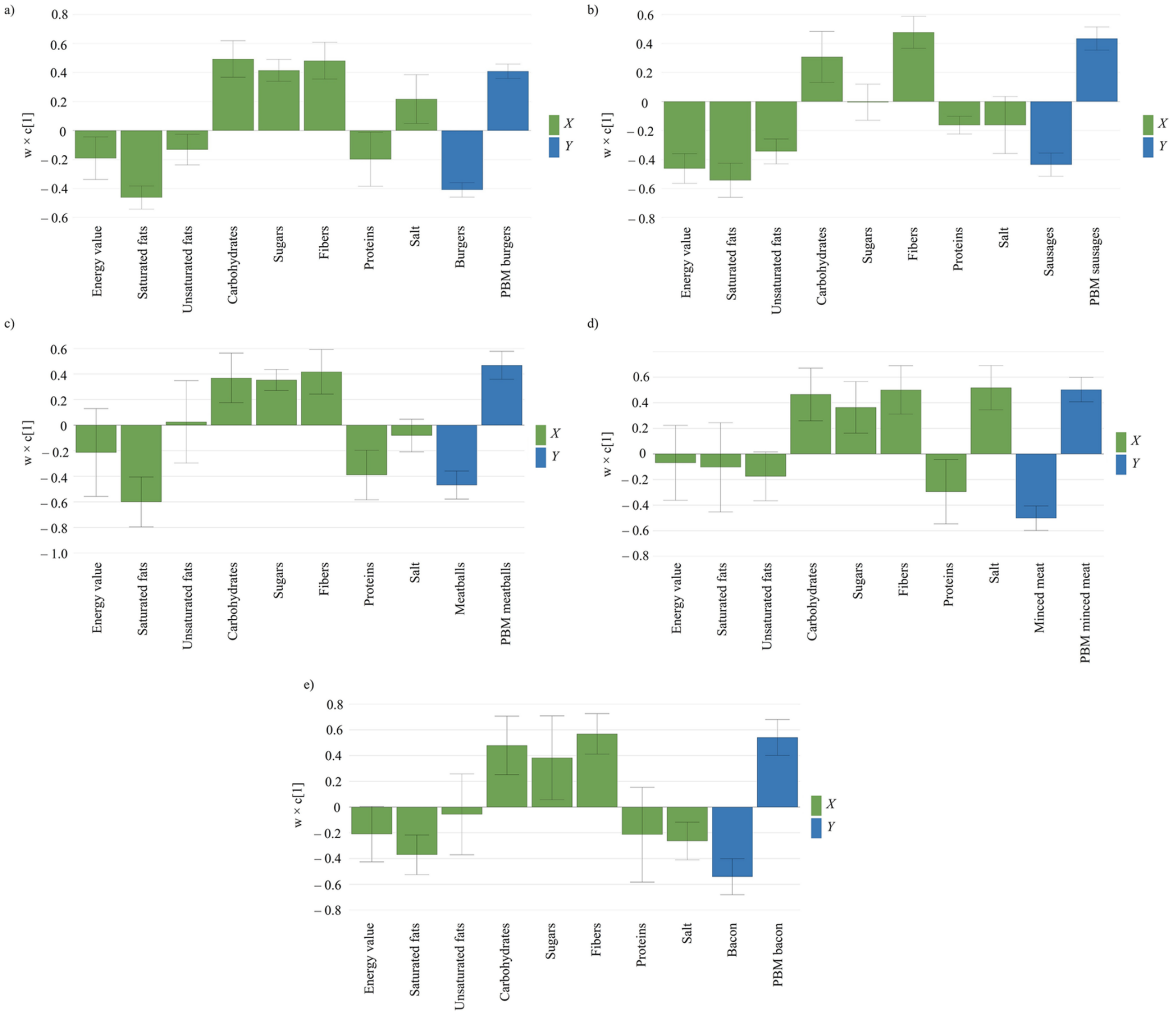
Loading plot illustrating the compositional differences between meat and PBM products. **(a)** burgers; **(b)** sausages; **(c)** meatballs; **(d)** minced meat; **(e)** bacon. The blue bars indicate the type of products in relation to their compositional particularities (green bars).

Comparing burger-type products between the two categories (Figure 2a), the patterns were like the results observed in the earlier meat vs. PBM product comparison. Meat products of these class had significantly higher energy values (*p* = 0.0017), which mainly derived from a markedly higher content in saturated fatty acids (*p* < 0.0001). The saturated fat content of meat products was around 2.5-times greater than their PBM counterparts. Although the unsaturated fatty acids content of meat products was higher compared to PBM products, this difference was not significant (*p* = 0.053). As such, not only differences in energy content, but also differences in lipid profiles can be addressed in terms of health concerns for these types of products. Regarding protein content, meat products were richer related to their PBM analogues.

The carbohydrate, sugar, and fiber content of PBM products was considerably greater when compared to their meat product counterparts (*p* < 0.0001). The fiber content in the case of PBM burgers was on average 4.0 g/100 g, compared to the 0.3 g/100 g observed in the case of traditional meat burgers. The differences are also emphasized by the maximal values observed for fiber content, as some PBM products contained as high as 13.0 g/100 g fibers, whereas in the case of meat burgers max. 1.5 g/100 g was noted. Intriguingly, the salt content of PBM burgers was higher, ranging from 0.7 to 4.3 g/100 g product, whereas salt content in the meat-based products ranged from 0.3 to 2.2 g/100 g product (*p* < 0.001).

#### 3.2.2 Sausages

Sausages obtained from meat were richer in both saturated (approx. 3-times) and unsaturated fatty acids (approx. 1.5-times), having a greater energy value than those manufactured from PBM (Figure 2b). The energy content differences are most pronounced in sausages, where PBM products contain nearly 100 kcal fewer per 100 g compared to their meat counterparts. Like burgers, PBM products offer not only a more favorable energy content, but also a better lipid profile, particularly in terms of lower saturated fats.

The sugar proportion driven from total carbohydrate content was similar between meat and PBM products (*p* = 0.378), although the carbohydrate content of PBM was noticeably higher than those of meat products. Contrary to burgers, meat sausages had more salt in their composition in comparison to PBM sausages (*p* < 0.05). The fiber content of PBM sausages also exceed, in a noteworthy manner, those prepared from animal meat. The fiber content of PBM sausages is comparable with those of burgers with an average fiber content of 3.5 g/100 g, compared to meat sausages whose fiber content is only 0.4 g/100 g.

#### 3.2.3 Meatballs

The meatballs manufactured from different sources showed a great difference in terms of saturated fatty acids (*p* < 0.0001) although the unsaturated fatty acids and energy values were comparable between the two types of meatballs, with *p* = 0.794 and *p* = 0.059, respectively (Figure 2c). Once again, a more optimal lipid composition is tilted toward the PBM products as comparable unsaturated fat content and noticeably lower saturated fat content is present in these products, which is also reflected in the energy values. As in the former cases, PBM meatballs were more abundant in carbohydrates, sugars, and fibers, whilst classical meatballs had a higher protein content. Nevertheless, even though PBM products contain on average about six times more fibers, some traditional meat products are more abundant in this nutrient, the maximal values between the two categories being comparable to each other. The salt content did not show noticeable differences between the two classes (*p* = 0.183).

#### 3.2.4 Minced meat

In terms of lipid content, minced meat derived from animal or plant sources did not show significant differences in saturated (*p* = 0.295) and unsaturated (*p* = 0.074) fatty acids, or in energy value (*p* = 0.485). Among the analyzed products, minced meat is the only one that did not show conspicuous differences between PBM and meat products, the energy values being a negligible 13 kcal/100 g product. Among the rest of the observed variables, carbohydrates, sugars, fibers, and proteins followed the patterns to those observed in the case of burgers, sausages and meatballs. The salt content was significantly higher in the case of PBM minced meat (Figure 2d).

#### 3.2.5 Bacon

Regarding unsaturated fatty acids content and energy value, no significant difference was observed between bacon of animal or plant origin, only the saturated fatty acids content was considerably higher in the case of meat products. The difference in protein content was borderline from a statistical point of view (*p* = 0.05). Otherwise, the carbohydrate, sugar, and fiber contents were markedly higher in PBM type products than in their meat equivalents (Figure 2e). Intriguingly, meat bacon was the only meat-based product with minimal to no fiber content, whereas the PBM type products were in the range of other products analyzed so far.

### 3.3 Quality indicators of the products other than the nutritional composition

Different food additives may influence the quality of the products, and it is mandatory that these substances, if present, to be written on the labels. Several indicators were found either exclusively or in greater quantities in meat products and here we are referring to additives such as sodium monoglutamate and sodium nitrite, although we have identified “other preservatives” in all PBM products.

Nutrients only found in PBM included minerals and vitamins. Regarding the natural and artificial color additives, they are not presented in the meat products, they were identified only in 12.5% of the burger-type products, on the other hand, all PBM products contain natural color additives and a small part of the sausages (9.8%), and bacon (33.3%) also contain artificial color additives (Table 3). Apart from these additives that are used in products for technological purposes, all the analyzed products, except for minced meat, contain “other additives,” influencing the final quality of the product.

**Table 3:** Micronutrients and food additives identified in meat and PBM products.

Products	Minerals, %	Vitamins, %	Nitrites, %	Other preservatives, %	Monosodium glutamate, %	Natural color additives, %	Artificial color additives, %	Other additives, %
PBM products								
Burgers (*n* = 46)	8.7	10.9	–	4.3	–	37.0	0.0	80.4
Sausages (*n* = 41)	12.2	19.5	–	31.7	–	29.3	9.8	85.4
Meatballs (*n* = 45)	8.9	8.9	–	4.4	–	22.2	0.0	75.6
Minced meat (*n* = 16)	6.3	6.3	–	6.3	–	75.0	0.0	93.8
Bacon (*n* = 12)	16.7	33.3	–	16.7	–	25.0	33.3	58.3
Meat products								
Burgers (*n* = 48)	–	–	2.1	33.3	4.2	12.5	0.0	50.0
Sausages (*n* = 50)	–	–	12.0	8.5	8.0	9.0	0.0	22.5
Meatballs (*n* = 25)	–	–	0.0	20.0	0.0	0.0	0.0	56.0
Minced meat (*n* = 24)	–	–	0.0	0.0	0.0	0.0	0.0	0.0
Bacon (*n* = 23)	–	–	82.6	26.1	0.0	0.0	0.0	87.0

### 3.4 Descriptive statistics and correlation of variables according to country

**Figure 3:**
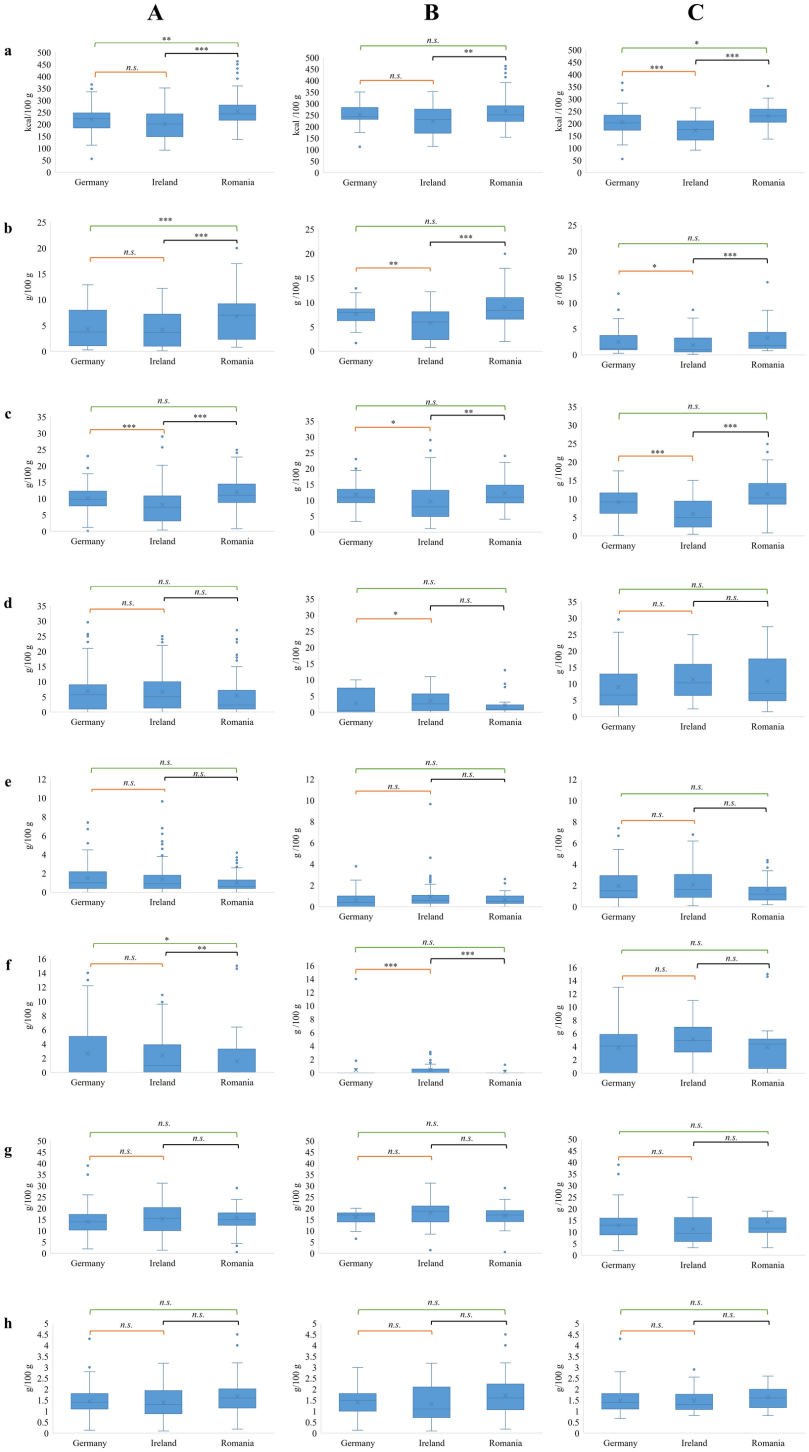
Box and Whiskers plots showing nutritional patterns between the analyzed countries. **(A)** All products; **(B)** meat products; **(C)** PBM products; **(a)** energy values, **(b)** saturated fats, **(c)** unsaturated fats, **(d)** carbohydrates, **(e)** sugars, **(f)** fibers, **(g)** proteins, **(h)** salt. n.s.—not significant, *p < 0.05, **p < 0.01, ***p < 0.0001.

The analysis of different markets from the perspective of energy values leads us to the conclusion that the products from Romania have a significantly higher energy value when compared to the products obtained from Germany and Ireland, whereas these latter two markets show no difference in terms of energy value (Figure 3Aa). Analyzing separately meat and PBM products, in the case of meat products, significant differences can be observed only between products from Ireland and Romania (Figure 3Ba). Regarding PBM products, the energy values show a noticeable market-to-market variation (Figure 3Ca). This difference is driven from the higher saturated fatty acids and unsaturated fatty acids content of the food products that can be found on the Romanian market compared to Germany and Ireland (Figures 3b,c).

In terms of carbohydrate (Figure 3d), sugar (Figure 3e), and protein content (Figure 3g), there was no significant difference between the three selected markets. The products obtained from the Romanian market are inferior in fiber content to those marketed in Germany and Ireland (Figure 3f). The salt content only differs between the products from Ireland and Romania, the products marketed in Romania having a higher content of salt (Figure 3h).

## 4 Discussion

This comparative study of the nutritional quality of PBM alternatives versus conventional meat products across three European markets (Romania, Germany, and Ireland) show that PBM products generally have lower energy density, significantly reduced saturated fat, and markedly higher fiber content compared to their meat counterparts. However, PBM products tend to contain lower levels of protein and, in several categories, higher salt content. We also observed notable regional differences: products available on the Romanian market displayed less favorable nutritional profiles, including higher energy, fat, and salt content, and lower fiber levels than those in Germany and Ireland. Our findings demonstrate the need for improved PBM formulations and legal framework alignment across countries. In the following sections, these results are discussed in relation to existing literature and broader public health implications

### 4.1 Market analysis of PBM in different countries

The present study aims to enhance awareness and deepen the understanding of nutritional disparities between PBMs and animal-based products while mapping variations across Eastern, Central, and Western European markets.

According to Eurostat, in 2022, Ireland ranked as the most economically developed country in the EU, Germany also demonstrated a high level of development. In contrast, Romania has a lower economic development level, being below the EU average. When considering Actual Individual Consumption (AIC per capita), which reflects actual household consumption and well-being, Germany ranked highest (117), followed by Ireland (94). Romania scored 86, further confirming a significantly lower standard of living compared to more developed EU countries. This discrepancy may limit the consummation of high-cost products for the general population in Romania, which can influence market demand and product availability (41). The German retail market for plant-based meat (PBM) alternatives is the largest in Europe, with sales continuing to expand, increasing by 42% between 2020 and 2022 to reach €1.91 billion. In contrast, the Romanian plant-based food retail market remains one of the smallest in Europe. However, sales data indicate a rapid rise in consumer demand, with sales of plant-based meat alternatives growing by 67% during the same period, reaching 163.5 million lei according to GFI (Good Food Institute) Europe (42). The noteworthy differences in PBM consumption across countries can be attributed, in part, to their relatively high price, which is directly correlated with consumers’ purchasing power and the availability of these products in retail stores. The Romanian market offers a more limited selection of PBM products compared to other countries; a fact confirmed during our product selection process for this study. In Romania, the range of available PBM products is considerably narrower, with many items sourced from national producers. In contrast, in the other two countries analyzed, consumers have access to a broader variety of products, including those from major international brands in this segment. From an economic perspective, higher income and education levels generally correlate with greater openness to new food products. North America and Europe are the largest markets for alternative proteins, yet disparities exist within Europe, particularly between Germany and Romania, due to differences in income, attitudes, knowledge, and religious influences (43). Religion plays a key role in Romania, where a predominantly religious population may be less open to food innovations. Additionally, studies suggest that politically liberal individuals are more inclined to purchase plant-based products, which could further explain Romania’s slower adoption rate in contrast to more progressive Western European countries (44, 45).

Recent studies on consumer perceptions of sustainability in food products reveal a growing awareness and interest in sustainable choices, though consumer understanding remains inconsistent. Key factors influencing perception include environmental impact, ethical sourcing, and health benefits. Clear labeling, recognizable certifications, and transparent communication from producers significantly enhance consumer trust and drive sustainable purchasing decisions. These findings highlight the need for standardized definitions and effective messaging to align consumer expectations with actual sustainability practices in the food industry (46–48).

Further interdisciplinary research is needed to assess not only the long-term health effects of consuming highly processed plant-based products but also how regulatory policies, consumer preferences, and economic constraints influence product development. Such investigations could support the creation of evidence-based guidelines to improve the nutritional quality, accessibility, and sustainability of plant-based alternatives globally. To enhance consumer acceptance, the involvement of food manufacturers and policymakers is essential in shaping the PBM market both in Romania and across Europe. Research and development efforts should focus on improving the sensory properties of PBMs to appeal to a wider demographic, particularly in regions where meat consumption is deeply embedded in culinary traditions.

### 4.2 Nutritional parameters of the products

The substantial differences in energy values and nutritional composition across markets emphasize the need for targeted interventions to address regional disparities in dietary choices and encourage healthier eating habits. Additionally, the observed variations in fiber content and salt levels among products from different markets highlight the importance of comprehensive strategies aimed at enhancing the nutritional quality of food products and improving public health outcomes on a global scale.

It is currently acknowledged, that, overall, PBMs contain lower protein levels, a modifiable fat content (notably devoid of cholesterol), and higher amounts of carbohydrates and dietary fiber compared to traditional meat products (49). Costa-Catala et al. examined the nutritional profiles of meat products and plant-based meat (PBM) alternatives available in Spain. Their findings indicated that PBMs typically had lower energy content, primarily due to reduced fat levels, and were richer in dietary fiber and complex carbohydrates. However, these alternatives generally contained less protein than traditional meat products and included a higher number of added ingredients (50). In another packaging evaluation study conducted in the UK by Ciobotaru et al., found that PBMs and conventional meat products had comparable energy content, despite PBMs having a higher total fat content. Additionally, while PBMs were richer in carbohydrates and dietary fiber, their protein content was notably lower compared to traditional meat products. (51). The study by Gréa et al. analyzed the nutritional content of PBMs compared to traditional meat products in Germany using PCA for comparison. The study found that PBMs generally contain less fat and saturated fat but more carbohydrates and sugars. Protein levels in PBMs were often comparable to or higher than in meat, especially in protein-based alternatives like sausages and salamis, however it was disclosed that more protein-based, rather than vegetable-based products were included in the analysis. While salt content varied, PBMs salamis had notably lower levels than their meat counterparts (52). Our results align with existing literature, as the analyzed meat products contained higher levels of both saturated and unsaturated fats, contributing to greater energy values compared to PBM products. Additionally, the protein content in meat products was higher than that of PBM alternatives. In contrast, PBM-type foods contained greater amounts of carbohydrates and fiber compared to their meat counterparts.

#### 4.2.1 Energy value

Regardless of the country, the global dietary trend is shifting toward reducing saturated fat intake and lowering obesity rates by promoting the consumption of nutrient-dense foods with lower energy content (53). Our findings reveal a statistically significant difference in the energy values of the two product groups analyzed, with meat products exhibiting a higher caloric content.

This disparity is also evident within the same product category across different countries. In Romania, PBM products available in stores have a higher average energy value (229 kcal/100 g) compared to those in Germany (205 kcal/100 g) and Ireland (172.6 kcal/100 g). These findings align with existing literature, which reports average energy values for PBM products ranging between 215 kcal/100 g and 240.7 kcal/100 g (3, 38).

The energy density of PBM products was significantly lower in the case of burgers and sausages, with all PBM categories exhibiting a lower energy value compared to their meat-based counterparts. However, despite their reduced caloric content, PBM products must still be classified as processed foods with a high energy value. According to Regulation (EC) No 1924/2006 of the European Parliament and of the Council of 20 December 2006 on nutrition and health claims made on foods, a product can only be labeled as “low energy” if it contains no more than 40 kcal/100 g (54).

Nevertheless, replacing conventional meat products with PBM alternatives may support weight management (55). This is supported by the results of a randomized crossover trial which showed that participants who consumed PBM instead of traditional meat lost weight. This is substantiated by a randomized crossover trial in which participants who consumed PBMs instead of traditional meat experienced weight loss (56). Similarly, a randomized controlled trial involving adults who followed a diet including unlimited meat substitutes for 4 weeks demonstrated a reduction in body weight. The study concluded that plant-based alternatives generally have a lower caloric density, which, when consumed over the long term, may contribute to sustained weight loss (57).

#### 4.2.2 Saturated and unsaturated fat

Our results indicate that saturated fat content was significantly lower in plant-based products, except for the minced meat category, where no significant differences were observed between plant- and animal-based products. These findings align with an Australian study reporting that plant-based burgers and sausages contain lower levels of saturated fat compared to their animal-based counterparts (4). The World Health Organization (WHO) has issued guidelines urging countries to implement population-level strategies to reduce saturated fat intake, aiming to lower the risk of non-communicable diseases, particularly cardiovascular diseases, in both adults and children (58). One such strategy is the substitution of animal meat with plant-based protein alternatives, which can contribute to reducing saturated fat consumption and, consequently, cardiovascular disease risk.

According to “Nutrition claims authorized in the annex of Regulation (EC) N° 1924/2006” products considered to have “low saturated fat content” are products in which saturated fatty acids are <1.5 g/100 and should not provide more than 10% of the energy value, with values between 1.5 and 5 g/100 g being considered products with a moderate content and over 5 g/100 g with a high content of saturated fat. The PBM products analyzed in this study exhibited an average saturated fat content of 2.5 g/100 g, classifying them as products with a moderate fat profile. In comparison, their meat-based counterparts demonstrated a markedly higher average saturated fat content of 7.4 g/100 g, positioning them within the highest category of saturated fat levels and thus indicating a less favorable nutritional profile.

Our results are comparable to those of Rizzolo-Brime et al., with average values of 10.0 g/100 g for meat products and 1.30 g/100 g for PBM products, similar values were described by Pointke et al. with values between 0.92 and 3.40 g/100 g (3, 38). Regarding the unsaturated fat content, the average for meat products was 11 g/100 g and for PBM products it was 8.7 g/100 g product. Only one category of PBM products, namely the meatball type products, has a higher content of unsaturated fats compared to their analogues (38).

#### 4.2.3 Carbohydrates and sugars

Unlike meat products, which are rich in protein, plant-based meat (PBM) alternatives contain higher levels of carbohydrates and sugars. This difference can be attributed to the addition of carbohydrate-based ingredients, such as starch and flour, which enhance binding, texture, and stability in PBM formulations (59). The carbohydrate sources in most plant-based products from our study, are wheat in the form of flour or starch, rice flour, potato starch and tapioca starch. In only one product we identified sugar and in another one caramel sugar syrup, all the other contain as source of carbohydrates the ones mentioned above.

#### 4.2.4 Fibers

PBM products also exhibited significantly higher fiber content than their animal-based counterparts. Given that fiber intake remains below recommended levels in most European countries incorporating PBMs into the diet could help consumers meet daily fiber requirements (60). Consistent with our findings, Curtain and Grafenauer reported that PBM analogues have higher carbohydrate and dietary fiber content than equivalent meat products (4).

Moreover, a recent review by Marczak et al. highlights dietary fibers as key modulators of food functionality, improving gel and tensile strength, water and oil-holding capacity, hardness, and chewiness, which contribute to the enhanced textural and sensory properties of PBM products (61). Non-digestible fibers, collectively referred to as plantix, offer multiple physiological health benefits. As these complex structures are resistant to digestion by human enzymes, they undergo colonic fermentation by gut microbiota, producing short-chain fatty acids (SCFAs) such as propionate and butyrate. These SCFAs play a crucial role in reducing blood cholesterol levels by inhibiting hepatic cholesterol synthesis, limiting cancerous cell proliferation, and modulating gut microbiota composition. By lowering intestinal pH, SCFAs promote the growth of beneficial Bifidobacteria and Lactobacilli, while suppressing pathogenic bacteria, thereby contributing to a healthier gut microbiome (62).

#### 4.2.5 Protein

Our study confirmed that meat products contain higher protein levels than their plant-based counterparts, aligning with previous research indicating lower protein content in PBMs compared to animal product (4, 36). Similarly, a UK cross-sectional survey found that four PBM categories had lower protein content than equivalent meat products. However, a study conducted in Spain reported different findings, concluding that plant-based meat analogues and animal meat products contain nearly identical protein amounts (63).

According to the European Food Safety Authority (EFSA), the recommended protein intake for healthy adults is 0.66 g per kg body weight per day, which equates to 46.2 g/day for a 70 kg adult (64). Our findings show that PBMs contain an average protein value of 12.8 g/100 g, indicating that these products can contribute substantially to daily protein intake. Beyond quantity, protein quality is critical, particularly in terms of essential amino acid composition. Yang et al. demonstrated that PBMs exhibit lower protein digestibility and release fewer bioactive peptides post-digestion (49). The limiting amino acid content of PBMs varies significantly from that of animal meat, particularly in lysine (Lys), threonine (Thr), and methionine (Met). To mitigate these deficiencies, modern PBMs incorporate diverse plant protein sources to enhance essential amino acid intake (16, 65). Commonly used plant protein sources include cereals (wheat, rice, barley, oats, spelt, corn), pseudocereals (quinoa, buckwheat), and legume-derived proteins (chickpeas, soy, mushrooms, lentils, lupins, peas, and beans) (3, 38, 66, 67).

#### 4.2.6 Salt

The salt content of PBM burgers ranged from 0.67 to 4.30 g/100 g, meaning their consumption can contribute to exceeding the 5 g/day limit recommended by the World Health Organization (WHO). Previous studies have reported similar findings regarding the high salt content of PBMs (3, 4). Our analysis revealed significantly higher salt levels in five PBM categories compared to their meat-based counterparts.

Excessive salt intake is a well-established risk factor for hypertension and cardiovascular diseases, while reducing dietary salt is recognized as a cost-effective public health strategy (68). Therefore, further efforts are needed to reduce salt levels in both plant-based and meat-based products.

#### 4.2.7 Other quality indicators

The nutritional quality of PBM and meat products depends on their ingredients and degree of processing. PBMs benefit from extensive fortification with minerals and vitamins (calcium, iron, folate, riboflavin, vitamin B12, magnesium, etc.), which can help prevent deficiencies, particularly vitamin B12 in vegetarians (8, 69, 70). However, meat remains a superior source of bioavailable iron, zinc, and potassium, even if these minerals are not explicitly listed as ingredients. A key distinction between the two categories is the use of preservatives. Sodium nitrite, widely used in processed meat for microbial safety, shelf-life extension, and color enhancement, has raised health concerns due to its potential conversion into carcinogenic N-nitroso compounds (71). Nitrites play a crucial role in processed meat by enhancing safety, shelf-life, and color, but their conversion into carcinogenic N-nitroso compounds raises health concerns, leading the EU to impose restrictions (72). In our study, all meat products except minced meat contained sodium nitrite, with bacon showing the highest prevalence (82.6%), whereas PBM products do not require nitrites but contain other preservatives.

Monosodium glutamate (MSG), a flavor enhancer, remains scientifically unproven as harmful, yet consumer preference leans toward MSG-free products (73). In a 2017 review conducted by the European Food Safety Authority (EFSA) Panel concluded that glutamic acid and its salts (E 620–625), when used as food additives, do not pose a genotoxic or general toxicological concern at controlled exposure levels. However, the Panel highlighted that estimated exposures in certain population groups may exceed this ADI and, in some cases, reach levels associated with adverse effects in humans, thus warranting continued monitoring and potential risk management measures (74). In line with this, Zamfirescu et al. have reported that clinical evidence on the health implications of MSG are contradictory and many of the reported negative health effect of MSG have little relevance for chronic human exposure and are poorly informative as they are based on excessive dosing that does not meet with levels normally consumed in food products (75). However, in contrast, a large-scale prospective cohort study conducted by Hasenböhler et al. revealed statistically significant associations between higher MSG (E621) intake and increased risk of coronary heart disease, urging the re-evaluation of these food additives by EFSA (76). Croitoru et al. assessed glutamate concentrations in various food products on the Romanian market, finding that meat items like salami and ham contained between 0.14 and 2.16 g/kg of glutamate. In contrast, vegetable mixes (3.32 g/kg) and highly processed foods such as soup cubes (79.95–119.95 g/kg) had significantly higher levels—equivalent to the glutamate content of approximately 7 kg of sausages (77). Similarly, Rhodes et al. reported MSG levels in UK food products, with meat and meat products containing 0.03–0.81% MSG and miscellaneous items ranging from 0.33 to 8.70%, aligning with the Romanian data. In the current study, MSG was not listed as an ingredient in any plant-based meat (PBM) products, whereas it was disclosed on the labels of 4.2% of meat burgers and 8% of sausages, indicating its selective use as a flavor enhancer in conventional meat products (78). While monosodium glutamate remains a scientifically supported and widely used food additive, consumer skepticism and misinformation continue to shape public perception. There is a need for balanced public health messaging, improved labeling transparency, and further research into long-term consumption patterns, particularly in vulnerable subgroups.

Regarding color additives, natural ones were found in all PBM products and in meat burgers and sausages, whereas artificial colorants appeared only in PBM bacon and sausages, some of which may pose carcinogenic risks, cause hypersensitivities, gastrointestinal and respiratory disorders (79). In children may pose significant health risks by adversely influencing cognitive function, behavioral patterns, metabolic processes, and overall development (80–82). In the case of PBM products the imitation of the sensory attributes of meat, i.e., color is one of the essential sensory attributes that shape consumer acceptability and palatability of these food products (83). This requirement prompts the PBM market to include natural or artificial coloring agents in the product to achieve a consumer perception similar to the appearance of meat (84). This observation is supported by our findings, which indicate that a greater proportion of PBM products contain higher levels of natural and/or artificial coloring agents than their meat product counterparts.

Both PBM and meat products contain food additives, making nutritional quality comparisons complex. A Spanish study using the NOVA system classified all PBM products as ultra-processed foods (Group 4) (85). Despite PBM products’ better nutritional profile, their degree of processing and additive content, particularly those with potential health risks should not be overlooked when considering these products as viable alternatives to animal foods.

### 4.3 Strengths and limitations of this study

The main limitations of this study include its relatively narrow geographic scope. Although data were collected from three distinct socioeconomic regions in Europe, the findings may not be fully generalizable to areas with different dietary habits. Differences in food labeling regulations could also affect the accuracy and comparability of nutritional data; however, the study prioritized consistently available label information across all evaluated products.

Another limitation is potential product selection bias, as the supermarkets chosen for data collection may favor specific brands, potentially overlooking regional alternatives with distinct nutritional profiles. These supermarkets were selected due to their accessibility to a large segment of the population in each country, but their offerings may not represent the full diversity of available products.

Additionally, the study does not account for potential changes in nutritional value when products are prepared and consumed in typical ways, which could influence their overall health benefits or risks (86). While the study focuses on the nutritional composition of products as sold, it does not directly assess the long-term health effects of regular PBM consumption, though these aspects are discussed using existing literature.

Further limitation of this study includes its reliance on nutritional labeling data, which may not fully capture variations in product composition or processing methods. Additionally, the study does not assess the long-term health impacts of PBM consumption, an area that warrants further investigation.

Despite these limitations, the study provides a rigorous and balanced analysis, offering valuable insights into the nutritional characteristics of PBM products while identifying areas for future research.

## 5 Conclusion

This study provides a comprehensive comparison of the nutritional quality of PBM alternatives and traditional meat products across three European markets. Our findings indicate that PBM products generally offer a more favorable nutritional profile, characterized by lower energy values, reduced saturated fat content, and significantly higher fiber levels compared to conventional meat. However, PBM products also tend to have lower protein content and, in some cases, higher salt levels, which should be considered when formulating dietary recommendations.

Market-specific variations were evident, with products from Romania displaying a less favorable nutritional profile than those from Germany and Ireland. The higher energy, fat, and salt content of Romanian PBM and meat products suggests a need for enhanced regulatory oversight and improved product formulation. These findings highlight the importance of food policies that encourage the development of nutritionally optimized PBM products while maintaining affordability and accessibility for consumers.

As plant-based meat alternatives continue to gain market share, further research is needed to evaluate their long-term health effects and potential role in dietary guidelines. The widespread adoption of PBM products presents an opportunity to improve public health outcomes and promote more sustainable food choices. However, efforts should focus on optimizing their nutritional composition to maximize their health benefits while minimizing undesirable additives and excessive processing.

## Data Availability

The original contributions presented in the study are included in the article/Supplementary material, further inquiries can be directed to the corresponding author.
