# Supplementary material for: Comparative analysis of the nutritional quality of plant-based and traditional meat products across European markets
Source: Front Nutr. 2025 Jul 16;12:1603600. doi: 10.3389/fnut.2025.1603600 (PMC12307488; doi:10.3389/fnut.2025.1603600)
Supplement: Supplementary file 1 [file Table_1.docx]

Supplementary Material

**Supplementary Table 1.** Descriptive statistics of meat and PBM products

| Type of product | Statistical indicators | | Energy (kcal/100 g) | Saturated fats  (g/100 g) | Unsaturated fats  (g/100 g) | Carbohydrates  (g/100 g) | Sugars  (g/100 g) | Fibers  (g/100 g) | Proteins  (g/100 g) | Salt  (g/100 g) |
| --- | --- | --- | --- | --- | --- | --- | --- | --- | --- | --- |
| *Burgers*  *(n = 48)* | *Average* | | 219.3 | 6.8 | 8.7 | 2.3 | 0.6 | 0.3 | 18.8 | 1.0 |
|  | *Min* | | 114.0 | 0.8 | 2.0 | 0.0 | 0.0 | 0.0 | 0.5 | 0.3 |
|  | *Q1* | | 204.4 | 5.6 | 7.1 | 0.4 | 0.1 | 0.0 | 17.0 | 0.8 |
|  | *Median* | | 232.0 | 7.6 | 9.0 | 1.0 | 0.4 | 0.0 | 18.1 | 1.0 |
|  | *Q3* | | 246.0 | 8.4 | 10.3 | 3.5 | 0.6 | 0.4 | 20.0 | 1.2 |
|  | *Max* | | 302.0 | 11.0 | 23.5 | 11.0 | 4.6 | 1.5 | 28.0 | 2.2 |
|  | *IQR* | | 41.6 | 2.8 | 3.2 | 3.1 | 0.6 | 0.4 | 3.0 | 0.4 |
| *PBM burgers*  *(n = 46)* | *Average* | | 189.9 | 2.7 | 7.1 | 11.8 | 2.4 | 4.0 | 13.1 | 1.3 |
|  | *Min* | | 56.0 | 0.1 | 0.1 | 0.3 | 0.0 | 0.0 | 2.0 | 0.7 |
|  | *Q1* | | 148.0 | 0.7 | 2.5 | 6.3 | 1.0 | 2.1 | 5.5 | 1.0 |
|  | *Median* | | 186.0 | 1.5 | 7.4 | 11.6 | 2.2 | 3.8 | 11.0 | 1.2 |
|  | *Q3* | | 230.5 | 4.2 | 9.7 | 16.7 | 3.7 | 5.7 | 16.7 | 1.4 |
|  | *Max* | | 366.0 | 8.7 | 24.9 | 29.6 | 7.4 | 13.0 | 91.0 | 4.3 |
|  | *IQR* | | 82.5 | 3.5 | 7.2 | 10.4 | 2.7 | 3.6 | 11.2 | 0.4 |
| *p* | | | *0.0017* | *<0.0001* | *0.053* | *<0.0001* | *<0.0001* | *<0.0001* | *<0.0001* | *0.0007* |
| *Sausages*  *(n = 50)* | *Average* | | 295.0 | 9.4 | 15.5 | 3.3 | 1.1 | 0.4 | 14.5 | 2.0 |
|  | *Min* | | 151.0 | 1.4 | 3.1 | 0.0 | 0.0 | 0.0 | 1.4 | 0.1 |
|  | *Q1* | | 252.5 | 7.6 | 12.0 | 1.0 | 0.4 | 0.0 | 12.4 | 1.6 |
|  | *Median* | | 281.5 | 9.0 | 14.9 | 1.4 | 0.8 | 0.0 | 14.2 | 2.0 |
|  | *Q3* | | 336.3 | 11.2 | 18.3 | 5.0 | 1.2 | 0.5 | 16.7 | 2.3 |
|  | *Max* | | 463.0 | 17.0 | 29.0 | 11.0 | 9.7 | 3.1 | 29.0 | 4.5 |
|  | *IQR* | | 83.8 | 3.6 | 6.3 | 4.0 | 0.8 | 0.5 | 4.3 | 0.7 |
| *PBM sausages*  *(n = 41)* | *Average* | | 199.7 | 2.6 | 9.7 | 7.6 | 1.1 | 3.5 | 12.3 | 1.7 |
|  | *Min* | | 129.0 | 0.4 | 1.1 | 1.0 | 0.0 | 0.0 | 5.0 | 0.9 |
|  | *Q1* | | 173.0 | 1.0 | 5.4 | 4.4 | 0.6 | 1.8 | 8.9 | 1.3 |
|  | *Median* | | 190.0 | 1.8 | 10.0 | 6.5 | 0.9 | 3.8 | 11.7 | 1.8 |
|  | *Max* | | 290.0 | 8.7 | 22.7 | 25.0 | 5.4 | 7.5 | 26.0 | 2.8 |
|  | *IQR* | | 59.0 | 2.6 | 7.9 | 4.4 | 0.7 | 3.2 | 5.4 | 0.7 |
| *p* | | | *<0.0001* | *<0.0001* | *<0.0001* | *<0.0001* | *0.3780* | *<0.0001* | *0.0036* | *0.0262* |
| *Meatballs*  *(n = 25)* | | *Average* | 240.6 | 6.0 | 9.8 | 7.2 | 1.2 | 0.8 | 16.1 | 1.5 |
|  |  | *Min* | 117.0 | 0.9 | 1.1 | 3.0 | 0.4 | 0.0 | 6.5 | 0.6 |
|  |  | *Q1* | 208.0 | 4.5 | 7.5 | 5.7 | 0.5 | 0.0 | 13.9 | 1.4 |
|  |  | *Median* | 240.0 | 6.2 | 10.5 | 8.0 | 1.0 | 0.0 | 14.0 | 1.6 |
|  |  | *Q3* | 286.0 | 8.0 | 13.0 | 8.8 | 1.4 | 0.4 | 17.6 | 1.7 |
|  |  | *Max* | 295.0 | 8.7 | 14.5 | 13.0 | 3.8 | 14.0 | 25.0 | 2.2 |
|  |  | *IQR* | 78.0 | 3.5 | 5.5 | 3.1 | 0.9 | 0.4 | 3.7 | 0.3 |
| *PBM meatballs*  *(n = 45)* | | *Average* | 218.8 | 1.7 | 10.0 | 13.8 | 2.4 | 5.2 | 11.2 | 1.4 |
|  |  | *Min* | 140.0 | 0.4 | 3.3 | 2.0 | 0.7 | 0.0 | 5.1 | 0.8 |
|  |  | *Q1* | 198.0 | 0.9 | 8.5 | 6.5 | 1.5 | 2.3 | 7.6 | 1.2 |
|  |  | *Median* | 223.0 | 1.1 | 9.4 | 14.0 | 1.8 | 5.0 | 9.8 | 1.4 |
|  |  | *Q3* | 241.0 | 1.5 | 12.0 | 19.0 | 3.3 | 7.3 | 15.0 | 1.7 |
|  |  | *Max* | 304.0 | 8.0 | 17.6 | 30.0 | 6.8 | 15.0 | 22.0 | 2.2 |
|  |  | *IQR* | 43.0 | 0.6 | 3.5 | 12.5 | 1.8 | 5.0 | 7.4 | 0.5 |
| *p* | | | *0.0591* | *<0.0001* | *0.7944* | *0.0021* | *<0.0001* | *<0.0001* | *0.0002* | *0.1835* |
| *Minced meat*  *(n = 24)* | | *Average* | 209.3 | 6.0 | 8.6 | 0.5 | 0.2 | 0.1 | 18.9 | 0.2 |
|  |  | *Min* | 121.0 | 1.3 | 2.3 | 0.0 | 0.0 | 0.0 | 12.0 | 0.1 |
|  |  | *Q1* | 169.0 | 3.9 | 5.9 | 0.0 | 0.0 | 0.0 | 18.0 | 0.1 |
|  |  | *Median* | 220.5 | 6.1 | 7.8 | 0.4 | 0.2 | 0.0 | 19.0 | 0.2 |
|  |  | *Q3* | 236.3 | 7.9 | 11.0 | 0.5 | 0.4 | 0.0 | 20.1 | 0.2 |
|  |  | *Max* | 328.0 | 16.0 | 23.0 | 2.8 | 1.0 | 0.4 | 24.0 | 1.0 |
|  |  | *IQR* | 67.3 | 4.0 | 5.1 | 0.5 | 0.4 | 0.0 | 2.1 | 0.1 |
| *PBM*  *minced meat*  *(n = 16)* | | *Average* | 196.7 | 4.7 | 5.9 | 5.0 | 1.1 | 3.8 | 16.0 | 1.3 |
|  |  | *Min* | 111.0 | 0.2 | 0.8 | 0.9 | 0.0 | 0.0 | 11.0 | 0.8 |
|  |  | *Q1* | 164.8 | 1.2 | 2.5 | 1.9 | 0.5 | 3.2 | 14.2 | 1.0 |
|  |  | *Median* | 201.5 | 3.7 | 5.5 | 5.3 | 1.1 | 4.4 | 15.0 | 1.2 |
|  |  | *Q3* | 240.0 | 6.1 | 8.0 | 7.1 | 1.6 | 5.4 | 17.7 | 1.6 |
|  |  | *Max* | 274.0 | 14.0 | 12.6 | 10.0 | 3.5 | 6.7 | 22.0 | 2.5 |
|  |  | *IQR* | 75.3 | 4.9 | 5.5 | 5.2 | 1.1 | 2.2 | 3.6 | 0.6 |
| *p* | | | *0.4849* | *0.2953* | *0.0744* | *<0.0001* | *0.0001* | *<0.0001* | *0.0027* | *<0.0001* |
| *Bacon*  *(n = 23)* | *Average* | | 234.4 | 7.1 | 10.1 | 0.9 | 0.5 | 0.0 | 18.7 | 2.5 |
|  | *Min* | | 113.0 | 1.7 | 2.4 | 0.0 | 0.0 | 0.0 | 13.0 | 1.5 |
|  | *Q1* | | 188.0 | 2.8 | 6.1 | 0.5 | 0.3 | 0.0 | 15.6 | 2.4 |
|  | *Median* | | 212.0 | 5.7 | 9.6 | 0.9 | 0.5 | 0.0 | 18.0 | 2.6 |
|  | *Q3* | | 276.5 | 9.6 | 13.4 | 1.1 | 0.7 | 0.0 | 20.9 | 2.7 |
|  | *Max* | | 415.0 | 20.0 | 20.0 | 2.1 | 1.5 | 0.5 | 31.2 | 3.2 |
|  | *IQR* | | 88.5 | 6.9 | 7.3 | 0.6 | 0.5 | 0.0 | 5.4 | 0.3 |
| *PBM bacon*  *(n = 12)* | *Average* | | 181.8 | 1.1 | 9.2 | 5.6 | 2.0 | 4.8 | 14.5 | 2.1 |
|  | *Min* | | 92.0 | 0.1 | 0.4 | 0.0 | 0.0 | 0.0 | 4.4 | 1.2 |
|  | *Q1* | | 178.8 | 1.3 | 6.2 | 1.1 | 0.4 | 0.0 | 11.0 | 1.0 |
|  | *Median* | | 184.5 | 1.1 | 10.0 | 5.1 | 1.6 | 5.4 | 13.0 | 2.4 |
|  | *Q3* | | 216.5 | 1.4 | 10.5 | 7.0 | 3.0 | 6.0 | 17.3 | 2.4 |
|  | *Max* | | 232.0 | 2.2 | 19.3 | 12.4 | 5.4 | 7.6 | 35.0 | 2.9 |
|  | *IQR* | | 37.8 | 0.1 | 4.3 | 5.9 | 2.6 | 6.0 | 6.3 | 1.4 |
| *p* | | | *0.0548* | *<0.0001* | *0.8213* | *<0.0001* | *0.0002* | *<0.0001* | *0.0501* | *0.0298* |

*n – number of products, Q – quartile, IQR – interquartile range, p – statistical difference for nutritional values between PBM and meat products*
